# ER biogenesis without stress: how the ribosome receptor, p180, defines a developmental program beyond the UPR

**DOI:** 10.3389/fcell.2025.1682420

**Published:** 2025-09-19

**Authors:** Payam Benyamini, David I. Meyer

**Affiliations:** ^1^ Department of Biomedical Sciences, College of Medicine, Charles R. Drew University, Los Angeles, CA, United States; ^2^ Department of Biological Chemistry, David Geffen School of Medicine at UCLA, Los Angeles, CA, United States

**Keywords:** rough endoplasmic reticulum, ribosome receptor, unfolded protein response, P180, upr, endoplasmic reticulum vesicular trafficking, endoplasmic reticulum stress, rough ER

## Abstract

The endoplasmic reticulum (ER) plays a central role in protein and lipid biosynthesis, quality control, and secretion. While its functional roles are well characterized, the mechanisms underlying ER biogenesis remain less defined. Developmental transitions in secretory tissues such as liver, pancreas, mammary gland, and plasma cells illustrate the remarkable capacity to expand their ER network in response to physiological demand. Central to this process is the ribosome receptor p180, a vertebrate-specific integral ER membrane protein whose expression is both necessary and sufficient for rough ER proliferation. Studies in yeast first demonstrated that overexpression of membrane proteins, including HMG-CoA reductase and domains of p180, induces membrane proliferation, thereby establishing yeast as a tractable model for ER biogenesis. In mammalian systems, p180 uniquely links membrane protein expression with biosynthetic scaling, enhancing ribosome binding, mRNA stabilization, lipid biosynthesis, and Golgi biogenesis. Gain- and loss-of-function approaches in human monocytic THP-1 cells confirm that p180 is indispensable for establishing a high-capacity secretory cells phenotype, coordinating the transition from sparse to abundant rough ER and secretory output. Importantly, p180-driven ER proliferation occurs independently of the unfolded protein response (UPR), highlighting distinct yet complementary mechanisms of ER remodeling: p180 as a constitutive biosynthetic scaffold and the UPR as a stress-induced regulator. Together, these findings position p180 as a master determinant of secretory architecture, with implications for development, immunity, and disease. Understanding the molecular underpinnings of p180 function and its integration with lipid metabolism and translation control will advance both basic cell biology and therapeutic strategies targeting secretory dysfunction. Recent work also suggests that p180-mediated ER expansion is dynamically tuned to nutrient availability and growth factor signaling, further linking organelle biogenesis to cellular metabolism. Dysregulation of p180 expression or function may contribute to a variety of pathological states such as cancer, neuronal dysregulation, and atherosclerosis where ER homeostasis is disrupted. Due to its vertebrate-specific origin, p180 also represents an evolutionary concerved lineage that enabled the diversification of complex secretory systems. Ultimately, dissecting the molecular circuits that govern p180 function promises to refine our understanding of organelle plasticity and to identify novel targets for therapeutic intervention.

## The biogenesis of the endoplasmic reticulum

Since it was first visualized in the electron microscope 80 years ago, a considerable number of physiological processes has been ascribed to the endoplasmic reticulum (ER), many of which have recently been elucidated in molecular detail (Baumann and Walz, 2001). These include the biosynthesis, translocation and processing of secretory and membrane proteins (Rapopo and rt, 1992; Brodsky, 1997; Johnson and Van Waes, 1999; Rapoport et al., 1999; Lippincott-Schwartz et al., 2000; Agarraberes and Dice, 2001), the biosynthesis of lipids (Kent, 1995; Carman and Zeimetz, 1996; Vance and Vance, 2008; Vance and Vance, 2004), and quality control and destruction of misfolded and/or foreign proteins within the ER (Braakman and Bulleid, 2011; Brodsky and Skach, 2011; Koenig and Ploegh, 2014; Benyair et al., 2011; Hendershot, 2004a; Cherepanova et al., 2016; Braunger et al., 2018; Ju et al., 2008). On the other hand, surprisingly little is known about the way in which the ER first appears and then, in many cell types, extensively proliferates often spanning the cytoplasm from the cell’s nucleus outward to the cell periphery.

During the development of tissues such as pancreas, liver, mammary gland or maturation of B-lymphocytes into plasma cells, a cytoplasm virtually devoid of rough endoplasmic reticulum (ER) membranes becomes populated with characteristic layer upon layer of evenly spaced rough ER within a short interval late in differentiation (Dallner et al., 1966a; Dallner et al., 1966b; Dallner et al., 1966b; Pictet et al., 1972; Janossy et al., 1973; Shohat et al., 1973; Chen-Kiang, 1994; Slack, 1995; Gittes, 2009; Debas, 1997; Henry et al., 2019; Lebman and Edmiston, 1999). In the case of liver and pancreas this occurs around the time of the animal’s birth, in mammary gland in advance of lactation, and in B-lymphocytes when triggered by an antigen to terminally differentiate. Accordingly, a sequence of events occurs that begins with an initial set of signals that culminates in an orchestrated profusion of events leading to the synthesis of lipid bilayer components, as well as the translation and integration of ER luminal and membrane proteins.

Published reports reveal (O'Keefe et al., 2022) that the expression of certain membrane proteins triggers a proliferation of lipid bilayers whose appearance, abundance and even composition are at least reminiscent of, and in some cases identical to rough ER (Benyamini et al., 2009; Bian et al., 2011; Sriburi et al., 2004; Schuck et al., 2009; Saito and Imaizumi, 2018). One such inducer of rough ER membrane biogenesis in mammalian cells is the 180 kDa ribosome receptor (p180). Discovered in the early 1990s as an integral rough ER membrane protein with high affinity for ribosomes, published studies have characterized many aspects of the rough ER induction and secretion process in a yeast model system (Savitz and Meyer, 1990; Savitz and Meyer, 1993; Wanker et al., 1995; Becker et al., 1999; Cui et al., 2012; Hyde et al., 2002; Block-Alper et al., 2002; Koppers et al., 2024). However, much remains to be elucidated regarding p180 in mammalian cells. Accordingly, The characterization of ribosome receptor as a critical factor emphasizes the intricate relationship between membrane protein synthesis and endoplasmic reticulum biogenesis. Despite significant advances, particularly from model systems, further investigation into the regulation and function of p180 and related components in mammalian cells is essential to fully elucidate the mechanisms driving ER membrane proliferation and its adaptation to cellular demands.

## The yeast as a historical model system

Significant breakthroughs in our ability to study ER proliferation at the molecular level have been made through the development of model systems. The discovery of “induced” membrane proliferation in yeast by Jasper Rine’s group enabled yeast to emerge as viable and genetically tractable model system for attacking the problem of ER proliferation (Wright et al., 1988; Wright, 1993; Parrish et al., 1995; Wright et al., 1990). The work by Robin Wright’s group has continued this line of research that originated with her findings that overexpression of an ER membrane protein, HMG CoA reductase, induced the proliferation of “karmellae” (Parrish et al., 1995; Wright et al., 1990; Lum and Wright, 1995; Koning et al., 1996; Profant et al., 1999). Even though karmellae appear as smooth concentric perinuclear arrays of membrane, they are probably not *bona fide* ER in terms of composition. The ER is a dynamic array of membrane tubules present in all cells, whereas karmellae are perinuclear concentric stacks of smooth membranes that are typically induced through the expression of specific ER membrane proteins. Karmellae are artificially produced under stress conditions and are not natural is structure. Their architecture is associated with alterations in lipid-protein balance rather than the structured cisternae, tubules and sheets that are standard to ER organization. They are restricted or have reduced capacity associated with ER functions, such as, protein folding and secretion of proteins into the extracellular space. Karmellae are believed to be adaptive ER domains formed under atypical conditions. Lastly, a main hallmark of a properly functioning ER is its association with ribosomes involved in secretory protein synthesis. In contrast, Karmellae appear as a storage depot for excessive membrane proteins. Nonetheless, one can reasonably assume that their formation involves many of the components and processes that eukaryotic cells use during their terminal differentiation. Similar studies have been carried out by a number of groups with the same results: Expression of certain membrane proteins triggers a proliferation of lipid bilayers whose appearance, abundance and even composition are at least reminiscent of—and in some cases identical—to rough ER (Wright et al., 1988; Wright, 1993; Parrish et al., 1995; Wright et al., 1990; Lum and Wright, 1995; Koning et al., 1996; Profant et al., 1999; Menzel et al., 1996).

## The unique properties of p180, the ribosome receptor

**FIGURE 1:**
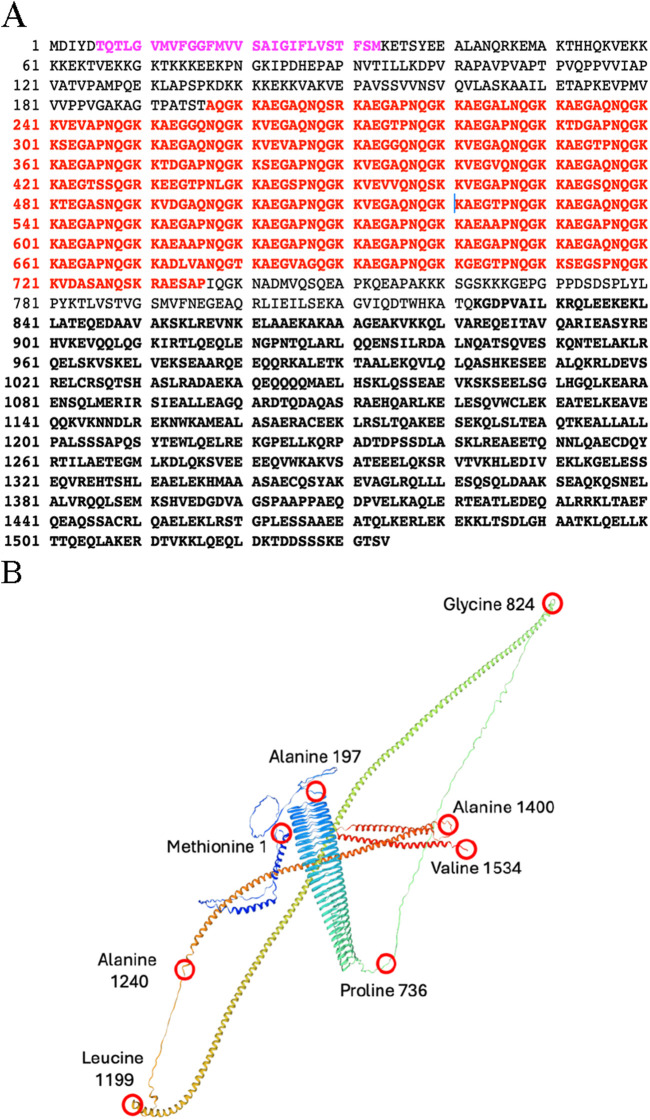
**(A)** Amino acid sequence of the Canine Ribosome Receptor (p180). Purple: Membrane anchor. Bold Red Repetitive domain is denoted in red. Bold Black: C-terminal domain is defined as amino acids 824–1534. And is predicted to contain up to four coiled-coil domains. **(B)** Structure of p180 predicted by Swiss-model algorithm with Global Model Quality Estimate of 0.71. Red circles represent amino acid positions. Membrane ancor: forms two short α-helices stretching from Methionine 1 to Alanine 197; Ribosome binding domain: forms a β-helix and stretches from Alanine 197 to Proline 736; the C-terminal domain forms several long α-helecies and stretches from Glycine 824 to valine 1534.

Thus far, p180 is the only protein whose expression has been shown to lead to increased levels of rough ER, elevated levels of ER luminal and membrane markers, and downstream components of the secretory pathway. Genes encoding canine, chicken, mouse and human p180 homologs have been identified, as well as homologs in other multicellular eukaryotes such as Zebrafish and *Xenopus*. Many findings include documenting a role for p180 in the efficient *in vitro* translocation of nascent polypeptides into intact and reconstituted mammalian ER (Profant et al., 1999). More importantly for ER biogenesis it was shown that the expression of distinct “domains” of p180 are capable of inducing membrane proliferation in yeast; the appearance and composition of the resulting membranes depending on the domain that was overexpressed (Wanker et al., 1995; Becker et al., 1999). The major subdivisions of p180 (Figure 1A) include an N-terminal hydrophobic sequence that anchors the protein in the ER membrane, a highly repetitive basic domain (pI = 11) having 54 uninterrupted, tandem repeats of the consensus decapeptide NQGKKAEGAP that forms an extensive ß-helix, which is necessary and sufficient for ribosome binding both *in vitro* and *in vivo*, and an acidic C-terminal domain (pI = 4.3) that is predicted to form coiled coils (Figure 1B) similar to those of known “cytolinkers” such as plectin (Liu et al., 1996). Expression of the N-terminal 151 amino acids of p180 (membrane anchor) was found sufficient to produce smooth, closely spaced, perinuclear membranes resembling the karmellae of Wright and Rine (Wright et al., 1988). The expression of constructs encoding the membrane anchor plus the ribosome binding domain, the membrane anchor and C-terminal domain, or the entire p180 molecule induced closely spaced rough membranes, smooth membranes with normal (100 nm) spacing, and rough membranes with 100 nm spacing, respectively (Becker et al., 1999).

A more detailed characterization of various aspects of this process has been achieved. Early studies in yeast indicated that the expression of the membrane anchored form of the repetitive N-terminal domain (involved in ribosome binding) 1) upregulated mRNA levels of proteins involved in membrane biogenesis and secretion, 2) led to a proliferation of functional rough ER, as well as other elements of the secretory pathway such as Golgi complexes, and 3) led to an overall increase in the ability of cells to secrete ectopically-expressed bovine pancreatic trypsin inhibitor (BPTI; (Becker et al., 1999)). These findings have been corroborated when it was determined that overexpression and induced expression of human p180 led to the upregulation of ER-ribosome docking, expression of secretory pathway specific proteins and biogenesis of secretory organelles (such as rough ER and Golgi) in mammalian cells (Benyamini et al., 2009; Ogawa-Goto et al., 2002; Ueno et al., 2010) validating the use of yeast as a model system. It should be noted that the expression of a p180 construct lacking the ribosome binding domain, like the expression of HMG CoA reductase, resulted in the proliferation of smooth membranes, without an increase in the abundance of mRNA encoding components of the secretory pathway (Becker et al., 1999; Block-Alper et al., 2002).

## The p180 ribosome binding domain forms an extensive β-helix that exhibits high affinity for ribosomes

**FIGURE 2:**
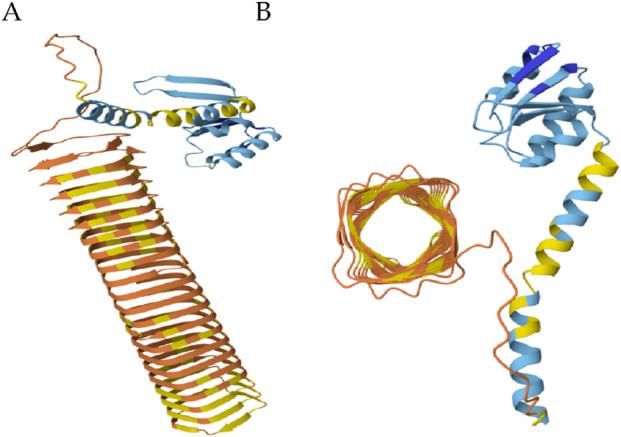
The ribosome binding domain forms a β-helix. **(A)** Side view of N-terminal domain. **(B)** Top view of the ribosome binding domain. Blue: membrane anchor; Orange and yellow: ribosome binding domain. (Global Model Quality Estimate of 0.72).

The N-terminal domain of p180 plays a critical role in its localization and function within the endoplasmic reticulum (ER). This domain encompasses distinct hydrophobic sequences that serve as membrane-targeting signals, facilitating the stable integration of p180 into the ER membrane (Becker et al., 1999; Block-Alper et al., 2002). These hydrophobic regions are not random but are structurally organized into coordinated segments comprising short stretches of α-helices interspersed with β-sheets, as revealed by high-resolution structural modeling (Figure 2). Such an arrangement is thought to help maintain both the stability and precise placement of the protein in the lipid bilayer, thereby securing its proper orientation for subsequent interactions.

Adjacent to the membrane-anchoring region lies a highly repetitive domain that mediates robust ribosome binding—a hallmark feature of p180s role in co-translational translocation and ER-associated protein synthesis. This domain is characterized by its extreme basicity (pI = 11) and remarkable structural repetitiveness, consisting of 54 uninterrupted tandem repeats of a conserved decapeptide motif (NQGKKAEGAP) (Wanker et al., 1995; Becker et al., 1999). Computational modeling indicates that this domain folds into an extended β-helix structure, forming a long, rigid scaffold that likely facilitates multivalent interactions with ribosomal subunits (Figure 2). Such a configuration could enhance the protein’s ability to tether multiple ribosomes simultaneously, effectively promoting the formation of polyribosomes on the ER surface. This organization not only amplifies the translational capacity of the ER but may also contribute to spatial organization of translation sites, particularly in cells with high secretory demands.

## Two key processes: p180 upregulation of lipid biosynthesis and stabilization of mRNA in yeast

Distinct morphologies and compositions of p180-induced membranes help dissect the mechanisms of membrane biogenesis. All p180 constructs promoted membrane and lipid bilayer proliferation, but only those with the ribosome-binding domain elevated mRNA and protein levels related to ER function and secretion (Becker et al., 1999). RNA microarray analysis showed significant upregulation of INO2, a master regulator of lipid and steroid biosynthesis, in all strains expressing p180-induced membranes, regardless of morphology (Block-Alper et al., 2002). Deletion of INO2 abolished membrane proliferation in response to known inducers, highlighting its essential role in lipid bilayer synthesis and providing a key entry point for studying membrane expansion in mammalian cells (Block-Alper et al., 2002).

A critical insight into how p180 supports a functional secretory pathway lies in its effect on mRNA stability. Yeast cells expressing p180 showed a 5–10-fold increase in the half-life of secretory protein mRNAs, closely tied to their association with p180-containing ER membranes (Hyde et al., 2002). This stabilization likely facilitates the upregulation of membrane protein components during biogenesis.

Although these findings resemble features of the unfolded protein response (UPR), studies in UPR-deficient yeast (IRE1 deletion; inositol-requiring enzyme 1) demonstrated that p180-induced mRNA upregulation and membrane proliferation occur independently of UPR activation (Becker et al., 1999). Thus, p180 appears to drive membrane biogenesis and secretory capacity through a distinct, UPR-independent pathway.

## p180 and the UPR are two distinct pathways that regulate ER function

Temporally and functionally, p180 and the UPR operate on fundamentally different timescales and cellular mandates. p180 serves as a constitutively active, long-lived facilitator of ribosome recruitment and ER-associated translation (Wanker et al., 1995; Becker et al., 1999). It is integral to maintaining high-throughput protein synthesis, particularly in cells with sustained secretory demands such as those in the pancreas, liver, kidney or plasma cells. p180s structural role in organizing polysomes at the ER membrane and expanding translational capacity supports continuous biosynthetic output and facilitates the enlargement of the ER membrane system to accommodate increased secretory load. (Benyamini et al., 2009; Wanker et al., 1995; Becker et al., 1999; Hyde et al., 2002; Ueno et al., 2010). Furthermore, published reports reveal that p180 associates with microtubules directly and possesses a novel microtubule binding domain, known as MTB-1. Overexpression studies of p180 in mammalian cultured cells induced acetylated microtubules in an MTB-1 depended manner (Ogawa-Goto et al., 2007). Additionally, Diefenback et al., has reported that the cytoplasmic coiled coil domain of p180, comprised of tandem repeats, contains a potential kinesin-binding domain downstream of MTB-1 (Diefenbach et al., 2004). It should be noted that p180 knockout in human monocytic cells (THP-1) shows ER morphological disruptions. Accumulation of vesicles and not cisternae (Benyamini et al., 2009). Suggesting, this is a feature of loss of interaction with the cytoskeleton. Specifically, the part of p180 that interacts with microtubules. It is possible that loss of this interaction leads to failure of vesicles to fuse into cisternae. Accordingly, these data reveal that p180s interaction with the cytoskeleton provides structural and functional integration between ER expansion, translation, and vesicular trafficking. It ensures that ER biogenesis is not just a matter of membrane supply but also of spatial organization and efficiency.

Conversely, the UPR is a dynamic, tightly regulated signaling network that is transiently activated in response to ER stress—specifically the accumulation of misfolded or unfolded proteins within the ER lumen (Hetz et al., 2020; Read and Schröder, 2021; Ron and Walter, 2007; Walter and Ron, 2011). This stress-responsive system initiates a multifaceted transcriptional and translational reprogramming designed to restore proteostasis (Acosta-Alvear et al., 2025; Karagöz et al., 2019). It does so by attenuating global protein synthesis (e.g., via PERK-mediated eIF2α phosphorylation) (Teske et al., 2011; Zhang and Kaufman, 2004), upregulating molecular chaperones (such as BiP/GRP78) (Ma and Hendershot, 2004; Hendershot, 2004b), enhancing ER-associated degradation (ERAD) pathways (Lemus and Goder, 2014), and in cases of unresolved stress, triggering apoptosis to eliminate dysfunctional cells (Fribley et al., 2009; Gardner et al., 2013).

While mechanistically distinct, these two systems can intersect, particularly in highly secretory cells. UPR activation, although initially antagonistic to protein synthesis, may facilitate longer-term adaptations that include the upregulation of p180. This upregulation serves as a compensatory mechanism during the recovery phase, allowing for ER expansion and resumption of high-output secretory activity once homeostasis is reestablished. Thus, p180 contributes to a post-stress rebuilding phase, anchoring the return to biosynthetic activity (Table 1).

**TABLE 1:** p180 and the UPR represent two distinct but occasionally convergent mechanisms of ER expansion. p180 and the UPR are functionally intersecting in high-secretory cells. Increased p180 expression may occur in parallel with or downstream of UPR activation to support ER expansion and increase the translational throughput once homeostasis is restored.

Attribute	p180	UPR
Nature	Structural ER membrane protein	Integrated signaling network
Cell Types	Hepatocytes, pancreas, plasma cells, kidney, acinar cells, alveolar epithelial cells, Goblet cells	All eukaryotic cells
Stimulus	Increased protein synthesis demand	Accumulation of unfolded/misfolded proteins
Function	Mediates ER biogenesis, ribosome docking	Restores ER homeostasis under stress
Mechanism of Action	Ribosome recruitment, lipid biosynthesis, cytoskeletal tethering	Transcriptional upregulation of chaperones, ERAD, translation attenuation
Pathway Dependence	UPR-independent	Involves IRE1, PERK, ATF6 pathways
Onset	Constitutive	Induced by unfolded or misfolded proteins
Physiological Context	Secretory differentiation to accommodate high translational load	Proteotoxic stress, hypoxia, nutrient deprivation
Cellular Role	Promotes protein synthesis and ER organization	Modulates translation, protein folding and degradation
Outcome	Biogenesis of rough ER to support secretion	Restoration of proteostasis, sometimes ER expansion as adaptation

While the ribosome receptor and UPR serve different purposes—biosynthesis vs. stress response—they are not mutually exclusive and can intersect. For instance, it is possible that during recovery from ER stress, UPR signaling may facilitate the upregulation of p180 or other translational machinery to restore secretory output once homeostasis is reestablished. Still, their core mechanisms, timing, and functional goals are distinct: the ribosome receptor enables continuous high-output protein synthesis, whereas the UPR transiently modulates ER activity in response to cellular conditions.

Taken together, p180 and the UPR represent complementary facets of ER function. p180 acts as a biosynthetic scaffold, optimizing and scaling translation at the ER, while the UPR functions as a sensor and regulator, preserving ER integrity under conditions of acute proteotoxic stress. Their coordinated interplay ensures that cells are equipped both to withstand stress and to resume or sustain the high-volume protein production required for physiological function.

## Human monocytes as a mammalian model system

Thus far, p180 is the only rough ER integral membrane protein whose expression leads to the establishment of a secretory phenotype (Benyamini et al., 2009; Ogawa-Goto et al., 2007). Genes encoding mouse and human p180 homologs have been identified, as well as homologs in other multi-cellular eukaryotes such as Zebrafish and *Xenopus*. However, no form of p180 exists in yeast, suggesting its function in tissue-specific processes exclusive to higher eukaryotes (e.g., vertebrates). In mammals the highest level of p180 expression is observed in tissues such as plasma cells, pancreas, placenta and liver, all of which have high levels of secretory activity. Published reports indicate that the differentiation of monocytes into macrophages is accompanied by the appearance of many of the hallmarks that constitute the process of terminal differentiation of mammalian progenitor cells into secretory cells (Benyamini et al., 2009; Tsuchiya et al., 1982).

These features are recapitulated in the THP-1 cell line (Tsuchiya et al., 1982). The ability of THP-1 cells to secrete Apolipoprotein E (ApoE) molecules involves extensive biogenesis of rough ER and upregulation of ER resident proteins involved in secretory protein synthesis, translocation, folding modification and trafficking to other organelles for further modification and secretion. The profound changes observed in THP-1 cells following induction and terminal differentiation into ApoE secreting macrophages has been well characterized (Benyamini et al., 2009; Tsuchiya et al., 1982); however, the intracellular events that stimulate rough ER membrane biogenesis and establishment of a secretory-cell phenotype had yet to be elucidated.

Using the human monocytic cell line (THP-1) we reported the results of studies that demonstrate the necessity of p180 in both the establishment of a secretory phenotype and accompanying increase in secretory capacity (Benyamini et al., 2009). Using different experimental modalities, we have shown the THP-1 cell can be induced to terminally differentiate and increase secretion upon stimulation by phorbol esters, notably 4β-phorbol-12β-mystrate-13α-acetate (TPA) (Benyamini et al., 2009; Tsuchiya et al., 1982). In response to TPA treatment this cell line upregulates p180, the production of rough ER membranes (Benyamini et al., 2009), and the synthesis and secretion of high levels of ApoE into the extracellular milieu (Benyamini et al., 2009). Morphological studies revealed that the TPA treatment of THP-1 cells led to adherence to the substrate and acquisition of an amoeboid shape (Benyamini et al., 2009). Comparisons between undifferentiated and terminally differentiated THP-1 cells showed a drastic change in overall intracellular structures associated with the establishment of a secretory phenotype, following TPA treatment (Benyamini et al., 2009; Tsuchiya et al., 1982).

## p180 expression levels increase disproportionally to rough ER biogenesis during terminal differentiation

To assess whether rough ER biogenesis is accompanied by a proportional increase in rough ER resident-protein expression levels before and after terminal differentiation, compared to p180 expression, DNA microarrays and Western blots were performed (Benyamini et al., 2009). Both experimental modalities displayed a proportional increase in calnexin and Sec61 levels with the overall increase of secretory pathway proteins, whereas assessment of p180 expression revealed that its mRNA and protein levels increased disproportionately, relative to the rough ER resident-proteins (Benyamini et al., 2009). This suggests that p180 may facilitate the establishment of a secretory phenotype. These observations provided good evidence showing that THP-1 cells serve as a suitable model system to investigate the underlying mechanism(s) associated with p180 mediated rough ER membrane biogenesis during the terminal differentiation of progenitor cells into high capacity, “professional”, secretory cells. Other examples of how protein fold-change affect mechanistic ordering of process drivers include a study where gene expression profiling characterizing B-cell differentiation revealed that ectopic expression of XBP1 induced the expression of secretory pathway genes that lead to secretory membrane expansion, while Blimp-1 deficient B-cells failed to upregulate majority of plasma cell-specific genes (Shaffer et al., 2004). Additionally, a landmark paper published by Rosen et al., showed that C/EBPβ and C/EBPδ are transiently elevated first during early differentiation, resulting in the induction of PPARγ and C/EBPα which reinforce each other to drive terminal adipogenic gene expression (Rosen et al., 2002). Lastly, a quantitative proteomics study tracing sequential waves of protein expression during myogenesis revealed time dependence in fold-changes correlated with cell cycle exit, RNA metabolism and the assembly of cytoskeleton associated myofibril structures (Le Bihan et al., 2015).

## Elucidating p180s function in mammalian cells using gene silencing

RNA interference (RNAi) using lentiviral vectors has proven to be a powerful tool for investigating protein function in mammalian cells (Brummelkamp et al., 2002; Paddison et al., 2002; Taxman et al., 2010). To investigate the functional significance of p180 in endoplasmic reticulum (ER) membrane proliferation and apolipoprotein E (ApoE) secretion, a targeted knockdown approach was employed using lentiviral shRNA constructs in THP-1 monocytes undergoing differentiation with TPA. Two stable cell lines were established for comparison: shRNA5, which harbored a scrambled, non-targeting sequence and served as a control for viral infection and selection, and shRNA6, which expressed a validated short hairpin RNA specifically targeting p180 mRNA (Benyamini et al., 2009).

Northern blot analysis confirmed the efficacy of the knockdown strategy. In shRNA6 cells, p180 transcript levels were reduced by approximately 75%–80% at 72 h post-TPA induction, while both shRNA5 and uninfected parental cells retained baseline expression levels of p180 mRNA. Notably, expression levels of PKCα, a kinase that mediates TPA-induced differentiation, were unaffected by shRNA transduction, indicating that all cell lines responded equivalently to TPA stimulation and that the observed effects were specific to p180 suppression (Benyamini et al., 2009).

Further temporal analysis showed that the reduction in p180 expression in shRNA6 cells was already established prior to TPA treatment and remained suppressed throughout the 72-h post-treatment window. This persistence of knockdown ensured that any phenotypic changes observed could be attributed directly to diminished p180 function over the entire course of differentiation and ER remodeling (Benyamini et al., 2009). Additionally, Ueno et al., employed small interfering RNA (siRNA) to knockdown p180 in human embryonic lung fibroblasts and showed a significant reduction in expression levels, further establishing the success of such strategy to study the functions of the ribosome receptor (Ueno et al., 2010).

These results validate the use of RNA-mediated silencing as an effective tool for modulating p180 expression in mammalian cells. Moreover, the model provides a robust and physiologically relevant system to dissect the mechanistic role of p180 in modulating ER biogenesis, structural expansion, and the secretory pathway—particularly its involvement in ApoE synthesis and trafficking during monocyte-to-macrophage transition, as well as collagen synthesis and secretion by human embryonic lung (HEL) cells, into the extracellular milieu (Ueno et al., 2010).

## p180 knockdown results in a decrease of rough ER biogenesis and levels of ER resident proteins

A significant decrease in rough ER is observed in p180 knockdown cells. Morphological assessment of shRNA-mediated knockdown of p180 in the THP-1 model system using multicolor immunofluorescence labeling in combination with morphometry measurements to quantify rough ER membrane biogenesis prior to and following TPA stimulation reveal a significant decrease in ER biogenesis. Mock infected, shRNA5 and shRNA6 expressing THP-1 cells were evaluated before and after TPA stimulation. Total surface area occupied by the rough ER and nucleus in the three unstimulated cells was essentially identical (Benyamini et al., 2009). Strikingly, following treatment with TPA, shRNA6 expressing THP-1 cells revealed a 68.7% decrease in rough ER surface area, compared to shRNA5 and mock-infected THP-1 cells. No significant change in ER surface area was observed between uninfected and shRNA5 expressing cells (Benyamini et al., 2009). These data suggest that terminally differentiated THP-1 cells expressing low levels of p180 exhibit a decreased capacity to proliferate rough ER membranes (Benyamini et al., 2009).

To confirm that the observed change in rough ER staining in shRNA6 cells is a result of decreased native proteins (calnexin) western blots were performed. Accordingly, western analysis comparing p180 and calnexin expression levels, per cell, revealed that shRNA6 cells synthesized 70% and 50% less p180 and calnexin protein, respectively, compared to controls (Benyamini et al., 2009). Taken together, it is evident that lowering endogenous p180 expression levels in terminally differentiated secretory cells leads to decreased biogenesis of rough ER membranes and synthesis of rough ER associated proteins.

## p180 knockdown leads to rough ER membrane fragmentation and decreased ribosome binding

Due to the limitations of immunofluorescence in determining membrane morphology Transmission Electron Microscopy (TEM) was employed to assess the consequences of p180 knockdown in the three terminally differentiated THP-1 cell lines (Benyamini et al., 2009). As expected, control cells exhibited extensive proliferation of evenly spaced rough ER cisternae, extending from the nucleus to the cell periphery (Benyamini et al., 2009). The ER membranes appeared studded with ribosomes and the distance between the membranes was observed to be approximately 100 nm. In contrast, electron micrographs of shRNA6 expressing cells revealed strikingly different membrane morphologies. Their membranes appeared highly vesiculated, not forming cisternae (Benyamini et al., 2009). A significant decrease in membrane-bound ribosomes is apparent in p180 silenced cells (Benyamini et al., 2009; Ueno et al., 2010).

To assess ribosome binding capacity of rough ER membranes in the uninfected, shRNA5, and shRNA6 expressing cells we used morphometric analysis to quantify the total number of bound ribosomes per unit area of membrane. Electron micrographs revealed that the vesiculated rough ER membranes of shRNA6 expressing cells bound significantly fewer ribosomes per unit area of membrane compared to control cells. Electron micrographs of shRNA6 cells further showed that in some cells the vesiculated membranes were completely devoid of ribosomes (Benyamini et al., 2009). Additionally, electron micrographs published by Ueno et al., showed that loss of p180 also lead to impaired association of ribosomes with the ER surface (Ueno et al., 2010). Examination of ribosome contents associated with ER membranes exhibited a high level of association with ribosomes in a p180 dependent manner (Benyamini et al., 2009; Ueno et al., 2010). In accordance with published reports, these observations provide evidence to suggest that loss of p180 leads to reduced ribosome binding to the ER membrane due to reduced p180 synthesis (Benyamini et al., 2009; Ueno et al., 2010), that results in an overall reduced stabilization of mRNA transcripts encoding for proteins that facilitate and maintain a fully differentiated secretory phenotype (Hyde et al., 2002). The vesiculated membrane morphology observed in electron micrographs of shRNA6 cells suggests that the state of the rough ER membrane system depends on adequate p180 expression levels. In terminally differentiated secretory cells, high p180 expression levels lead to increased rough ER membrane cisternae formation, whereas lower levels of p180 expression favor the formation of vesiculated rough ER membranes (Benyamini et al., 2009) with significantly lower membrane associated ribosomes (Benyamini et al., 2009; Ueno et al., 2010). Representative electron micrographs of 72 h TPA-treated THP-1 cells, show elongated rough ER cisternae with bound ribosomes. Representative electron micrographs of 72-h TPA-treated shRNA6 cells and siRNA transfected HEL cells show vesiculated ER membranes (Benyamini et al., 2009) virtually devoid of ribosomes (Benyamini et al., 2009; Ueno et al., 2010).

## p180 knockdown decreases ApoE secretion accompanied by accumulation of intracellular ApoE

To assess the impact of shRNA6 on the secretory capacity of THP-1 cells, ELISAs were performed to measure ApoE secretion into the culture medium. Both shRNA6 and control cells (mock-infected and shRNA5-expressing) were treated with TPA and incubated for 72 h. ELISA results showed a significant reduction in ApoE secretion specifically in shRNA6 cells, while control groups secreted ApoE at similar levels (Benyamini et al., 2009). As ApoE is synthesized in the rough ER and undergoes post-translational modification and trafficking through the Golgi network before secretion (Moon et al., 2024; Shuvaev et al., 1999; Flowers et al., 2020; Ke et al., 2020; Lee et al., 2010; Jaros et al., 2012; Raftery et al., 2005; Miyata and Smith, 1996; Huang and Wang, 2017; Kockx et al., 2018), the observed reduction could reflect defects in synthesis, processing, or transport. Similar observations were made when assessing procollagen synthesis, folding and post-translational modifications (Ueno et al., 2010).

To explore this, Western blot analysis was performed to measure intracellular ApoE. Results revealed a clear accumulation of ApoE in shRNA6 cells, suggesting impaired secretion rather than reduced synthesis (Benyamini et al., 2009). To localize this intracellular ApoE, cell fractionation was conducted following TPA treatment. Western blotting of the cytosolic fraction showed increased levels of unmodified ApoE in shRNA6 cells, while membrane fractions displayed elevated levels of both unmodified and secretory forms, compared to controls (Benyamini et al., 2009). These findings indicate that p180 knockdown disrupts multiple stages of the secretory pathway.

A likely explanation for the increased cytoplasmic ApoE is reduced mRNA stabilization and retention at the ER membrane, leading to translation in the cytosol. This is supported by electron microscopy showing a marked reduction in ribosome association with rough ER membranes in shRNA6-expressing cells (Benyamini et al., 2009). Lastly, published reports further reveal that small interfering RNA mediated knockdown of p180 in human embryonic lung cells induced a prominent reduction in extracellular matrix protein secretion, such as collagen and fibronectin, into the extracellular milieu (Ueno et al., 2010).

## p180 knockdown leads to decreased golgi biogenesis

The Golgi apparatus is situated geographically and functionally central in the secretory pathway between the rough ER and the plasma membrane. It is usually organized into series of flattened cisternae with surrounding tubules and vesicles. In addition to sorting and transporting newly synthesized ER-derived proteins, the Golgi also functions as post-translational modifier of glycoproteins and glycolipids (Huang and Wang, 2017). In the case of ApoE post-translational modifications, the Golgi is the organelle responsible for addition of is O-linked sugar sequences (Kockx et al., 2018). The observed accumulation of the secreted form of ApoE in shRNA6 cells, in conjunction with preliminary microarray results indicating significantly altered levels of mRNA transcripts encoding for phospholipid proteins, led us to speculate that the structural integrity of the Golgi apparatus may be compromised (Benyamini et al., 2009). To determine if, due to decreased levels of p180, the integrity of the Golgi complex is also affected, TEM was employed to assess the membrane morphology of the Golgi in mock-infected control, shRNA5 and shRNA6 cells.

Electron micrographs of the Golgi complex in control cells revealed the appearance of multiple tightly packed parallel arrays of cisternae with elongated and expanded peripheral structures (Benyamini et al., 2009). In contrast, the membrane morphology of the Golgi apparatus of shRNA6 expressing cells appeared vesiculated and in many cases highly unorganized. Fewer Golgi-like structures were observed in the cytoplasm of p180 knockdown cells compared to controls (Benyamini et al., 2009). These findings are consistent with our hypothesis suggesting that p180 expression is required for establishment of downstream components of the secretory pathway as well.

## p180 overexpression is sufficient for the establishment of a secretory cell phenotype

Owing to the intriguing finding that the membrane morphology of the rough ER p180-deficient THP-1 cells appeared vesiculated, compared to cisternae observed in controls, we hypothesized the p180 overexpression levels dictate this aspect of rough ER morphology. To address whether p180 expression induces the biogenesis of rough vesicles and proliferation of cisternae, we transiently transfected Human Embryonic Kidney 293 (HEK293) cells with mammalian expression vectors encoding full length p180 (Benyamini et al., 2009).

Electron micrographs of control untransfected HEK293 cells showed a cytoplasm virtually devoid of rough ER membranes and other downstream components of the secretory pathway, such as the Golgi complex (Benyamini et al., 2009). In contrast, electron micrographs of p180 transfected HEK293 cells revealed a significant increase in the biogenesis of the rough ER-like vesicles in the cytoplasm (Benyamini et al., 2009). It is apparent that these p180 induced rough vesicles are localized to the perinuclear membrane, as well as other pre-existing rough ER membranes in the cytosol. This suggests that these rough vesicles are either preparing for homotypic-fusion to cisternae and or have just undergone fragmentation from pre-existing, cisternal rough ER membranes into vesicles. Furthermore, the proliferation of rough membranes extending from the nucleus to the cell periphery was apparent as well. The spacing between the rough ER membrane cisternae appeared to be approximately 100 nm, the norm for secretory cell rough ER. Additionally, Ueno et al., reveal that elevation of the p180 levels by ascorbate stimulation or overexpression resulted in a concomitant increase in ER membranes associated with ribosomes (Ueno et al., 2010). Taken together, these studies show that the expression of p180 alone is sufficient to induce the biogenesis of rough ER-like vesicles and ribosomes bound membrane cisternae (Benyamini et al., 2009; Ueno et al., 2010).

## p180 overexpression leads to golgi complex biogenesis

In addition to the rough ER, the Golgi apparatus is another organelle that functions and resides in the middle of the secretory pathway, between the rough ER and the plasma membrane. In mammalian cells the Golgi complex represents a functionally polarized organelle through which newly synthesized lipids and proteins, exported from the rough ER, are further modified and prepared for trafficking to the plasma membrane for secretion and other organelles, such as peroxisomes and lysosomes for additional modifications (Huang and Wang, 2017; De Duve and Baudhuin, 1966; Lowe, 2002).

To determine whether p180 expression is sufficient to induce the biogenesis of Golgi complexes a non-secretory HEK293 model system was used. Electron micrographs of HEK293 cells transiently expressing wild-type p80 showed a significant increase in Golgi biogenesis (Benyamini et al., 2009). In contrast, control cells expressing a vector control showed no apparent increase in Gogi membranes (Benyamini et al., 2009). Taken together, our results provide evidence showing that overexpression of p180, in a non-secretory cell, leads to the proliferation of Golgi complexes as well as rough ER membrane biogenesis.

## The “crowded membrane” model

**FIGURE 3:**
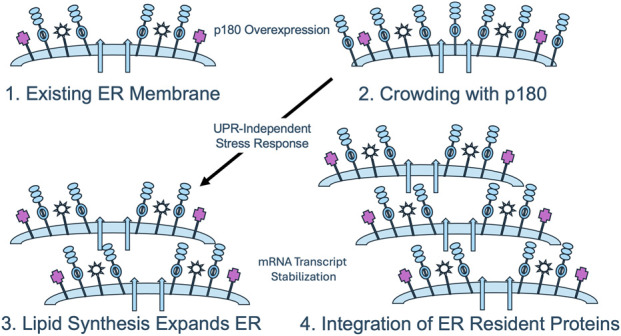
The crowded membrane model for p180-mediated ER proliferation. When p180 is overexpressed, existing ER membranes (1) become “crowded” through the insertion of molecules of p180 (2). A potential UPR-independent stress response upregulates lipid biosynthesis, allowing dilution of the lipid bilayer and expansion of the existing membrane (3). Concomitantly or subsequently, through p180-mediated stabilization of secretory pathway mRNAs, expansion of normal, functional rough ER is achieved.

Based on these findings, we propose the following working model for ER membrane proliferation that will enable testable hypotheses to be formulated for this proposal (Figure 3). It is based on the simple observation that the expression of high levels of p180 in both yeast and mammalian cells leads to proliferation of membranes that morphologically and functionally resemble those appearing during the terminal differentiation of mammalian secretory cells and tissues. The premise, therefore, is that the upregulation of p180 is necessary and sufficient to induce primary events leading to increased secretory capacity. We propose that the insertion of increased levels of p180 into existing ER membranes has the following two initial consequences: 1) The upregulation of lipid biosynthesis and assembly into ER membranes and 2) the selective stabilization of secretory pathway mRNAs insuring an appropriate level of synthesis and incorporation of ER secretory and membrane proteins. Upon sensing that the membrane has become overcrowded with newly-integrated proteins, a yet-to-be characterized mammalian stress response triggers the synthesis of new lipids that eventually organize into rough ER bilayers. At the same time ER proteins and components of the secretory pathway are translated at increased levels due to the mRNA stabilizing activity of p180 that has been incorporated into newly synthesized ER. A logical corollary to this theory is that the level of p180 expression determines the extent to which membranes are proliferated, something that is observed in mammalian cells.

## Conclusion

These studies identify the ribosome receptor p180 as a critical and evolutionarily recent determinant of secretory cell architecture. Through a combination of gain- and loss-of-function studies in yeast and mammalian cells, it has been demonstrated that p180 orchestrates multiple hallmarks of the secretory phenotype: ER membrane proliferation, mRNA stabilization, ribosome loading, and Golgi biogenesis (Benyamini et al., 2009; Wanker et al., 1995; Becker et al., 1999; Block-Alper et al., 2002; Ueno et al., 2010; Ogawa-Goto et al., 2007). These findings establish p180 not merely as a structural adaptor but as a master regulator of secretory capacity, acting at the intersection of membrane biology, translational control, and organelle morphogenesis.

In mammalian THP-1 monocytes undergoing TPA-induced differentiation, upregulation of p180 coincides with increased rough ER content, mRNA stabilization of secretory transcripts, and robust secretion of ApoE—a lipid-binding protein implicated in immune modulation and neurodegeneration. Knockdown of p180 in these cells disrupts each of these processes, leading to vesiculated ER membranes devoid of ribosomes, Golgi disorganization, and intracellular accumulation of secretory proteins (Benyamini et al., 2009; Ueno et al., 2010). These defects, which phenocopy an arrested secretory differentiation state, underscore the essentiality of p180 in coordinating the cellular transition from a non-secretory to a secretory phenotype.

The specificity of p180s effects is underscored by its sufficiency to reprogram a non-secretory cell. In HEK293 cells—normally characterized by sparse ER and negligible Golgi structure—transient expression of p180 alone induces extensive biogenesis of rough ER membranes and Golgi cisternae (Benyamini et al., 2009). These changes occur independently of exogenous differentiation cues or ER stress, suggesting that p180 operates upstream of canonical pathways that control organelle biogenesis.

It should be noted that p180 and the Unfolded Protein Response (UPR) are both implicated in the remodeling and expansion of the endoplasmic reticulum (ER), but they operate through fundamentally different biological paradigms—structural augmentation versus stress mitigation. p180 is a specific ER membrane-bound ribosome receptor and coiled-coil protein that plays a direct role in ribosome anchoring, translation-associated ER expansion, and biosynthetic scaling of the rough ER. Overexpression of p180 has been shown to induce ER membrane proliferation independently of protein folding stress (Block-Alper et al., 2002). It enhances the biosynthesis of ER membrane lipids and recruits ribosomes to the ER membrane, which is especially important during physiological processes requiring massive protein production, such as plasma cell differentiation and secretory tissue development. p180 acts as a structural scaffold that helps expand the rough ER network to accommodate increased translation demands (Savitz and Meyer, 1990; Savitz and Meyer, 1993; Wanker et al., 1995; Becker et al., 1999; Hyde et al., 2002; Diefenbach et al., 2004).

In stark contrast, the UPR is a cellular stress response mechanism activated by the accumulation of unfolded or misfolded proteins in the ER lumen (Walter and Ron, 2011; Acosta-Alvear et al., 2025; Karagöz et al., 2019). The UPR aims to restore homeostasis by upregulating chaperone proteins, enhancing ER-associated degradation (ERAD), temporarily attenuating translation, and promoting ER expansion if the stress persists (Lemus and Goder, 2014; Hwang and Qi, 2018). The UPR involves three major signaling pathways—IRE1, PERK (protein kinase R-like ER kinase), and ATF6 (activating transcription factor 6)—which collectively regulate transcriptional and translational changes (Read and Schröder, 2021; Ron and Walter, 2007; Gardner et al., 2013; Karagöz et al., 2017). While the UPR can lead to ER proliferation, it does so as a secondary response to stress, not as a primary driver of ER biogenesis under normal physiological conditions. p180 drives ER proliferation proactively in response to developmental or functional demands for increased protein synthesis, whereas the UPR is a reactive stress response pathway that indirectly contributes to ER expansion as part of its strategy to manage proteotoxic stress.

This model reconciles previously disparate observations: (i) that ER expansion can occur independently of stress pathways such as IRE1/XBP1 or PERK, (ii) that p180 overexpression is sufficient for Golgi biogenesis even in non-secretory cells, and (iii) that membrane crowding itself may serve as a physiological signal. Notably, our findings decouple ER expansion from traditional stress responses and link it instead to structural protein expression, suggesting that p180 may define a developmentally programmed pathway of organelle expansion, distinct from UPR mediated stress-induced remodeling.

The implications of this work are broad. The emergence of p180 in vertebrates—absent in yeast and most invertebrates—may reflect an evolutionary adaptation enabling dynamic control of secretory architecture in response to physiological cues, such as differentiation, inflammation, or regenerative demand. Dysregulation of p180 expression or function could contribute to diseases characterized by cancer (Tsai et al., 2013; Pan et al., 2015; Liang et al., 2015; Li et al., 2019; Chen et al., 2022), neurodegeneration (Koppers et al., 2024; Ogawa-Goto et al., 2007; Killackey et al., 2023; Kavanagh et al., 2022; Colla, 2019), atherosclerosis (Chiu et al., 2023; Wang et al., 2023), immunodeficiencies (The Human Protein Atlas), or metabolic syndrome (LANGLEY et al., 1998). Moreover, forced expression of p180 could be explored as a synthetic strategy to enhance protein production in biotechnological or regenerative contexts.

Taken together, these studies position p180 as a central regulator of the secretory pathway, capable of coordinating lipid synthesis, translational activity, and organelle morphology through a unified, p180-centered mechanism. Future research should define the molecular determinants of p180-mediated mRNA stabilization, identify the lipid biosynthesis pathways it recruits, and dissect the signaling mechanisms that detect membrane crowding. Together, these directions will refine the “crowded membrane” model and open new avenues for understanding and manipulating secretory cell biology.

## Data Availability

The original contributions presented in the study are included in the article/supplementary material, further inquiries can be directed to the corresponding author.
